# The Beneficial Effect of Coarse Cereals on Chronic Diseases through Regulating Gut Microbiota

**DOI:** 10.3390/foods10112891

**Published:** 2021-11-22

**Authors:** Guixing Ren, Xin Fan, Cong Teng, Yajie Li, Nadia Everaert, Christophe Blecker

**Affiliations:** 1 College of Pharmacy and Biological Engineering, Chengdu University, No. 1 Shilling Road, Chenglo Avenue, Longquan District, Chengdu 610106, China; Liyajie0303@Foxmail.com; 2 Institute of Crop Science, Chinese Academy of Agricultural Sciences, No. 80 South Xueyuan Road, Haidian District, Beijing 100081, China; fx_926@163.com (X.F.); 8101172124@caas.cn (C.T.); 3 Gembloux Agro-Bio Tech, University of Liège, 5030 Gembloux, Belgium; nadia.everaert@uliege.be (N.E.); christophe.blecker@uliege.be (C.B.)

**Keywords:** bioactive components, inflammation, hyperlipidemia, obesity, diabetes, short-chain fatty acids

## Abstract

In recent years, chronic diseases including obesity, diabetes, cancer, cardiovascular, and neurodegenerative disorders have been the leading causes of incapacity and death globally. Increasing evidence suggests that improvements of lifestyle habits and diet is the most commonly adopted strategy for the prevention of chronic disorders. Moreover, many dietary compounds have revealed health-promoting benefits beyond their nutritional effects. It is worth noting that diet plays an important role in shaping the intestinal microbiota. Coarse cereals constitute important sources of nutrients for the gut microbiota and contribute to a healthy gut microbiome. Furthermore, the gut microbiota converts coarse cereals into functional substances and mediates the interaction between the host and these components. In this study, we summarize the recent findings concerning functional components of cereal grains and their potential chemopreventive activity via modulating the gut microbiota.

## 1. Introduction

Coarse cereals are cereal grains except for rice and wheat, including oats (*Avena sativa*), buckwheat (*Fagopyrum esculentum*), barley (*Hordeum vulgare*), sorghum (*Sorghum vulgare*), quinoa (*Chenopodium quinoa*), and some millet species [1]. Due to their rich protein content and balanced amino acid profile, the nutritional value of coarse cereals has attracted wider attention than that of major cereals [2]. In addition to their nutritional value, the main bioactive substances (dietary fiber, starch, flavonoid, polyphenol, saponin, polysaccharide, protein, and peptides) of coarse cereals have been demonstrated to exert various biological functions. For instance, a previous study reported that coarse-cereal fiber had a positive effect on cardiometabolic and obesity-related diseases [3]. Idehen et al. found that barley phytochemicals (phytosterol, flavonoids, phenolic acids, etc.) can exert antioxidant, cholesterol-lowering, and anti-cancer functions to prevent cardiovascular diseases [4]. It has been reported that phytosterols play a crucial role in inhibiting the progression of cancers [5].

Dietary structure is a significant impact factor in intestinal microecology, which demonstrates a trend towards dynamic change along with changes in eating habits and trophic factor intake [6]. The roles that human colonic bacteria or the gut microbiota play in health and disease have received considerable attention over the last decade, supported by recent reports suggesting that the gut microbiota have the potential to treat many diseases that plague modern society, including obesity, diabetes, and colorectal cancer [7]. As previously described, the main microorganisms in the human intestine are closely related to the nutritional components and their proportions in the dietary structure [8]. A diet rich in fiber stimulates an increase in the number of *Bifidobacteria* in the human intestine [9]. Moreover, a high consumption of protein and fat is responsible for *Bacteroides*, and high saturated fatty acids (FAs) can promote an abundance of *Bilophila wadsworthia* [10].

**Figure 1:**
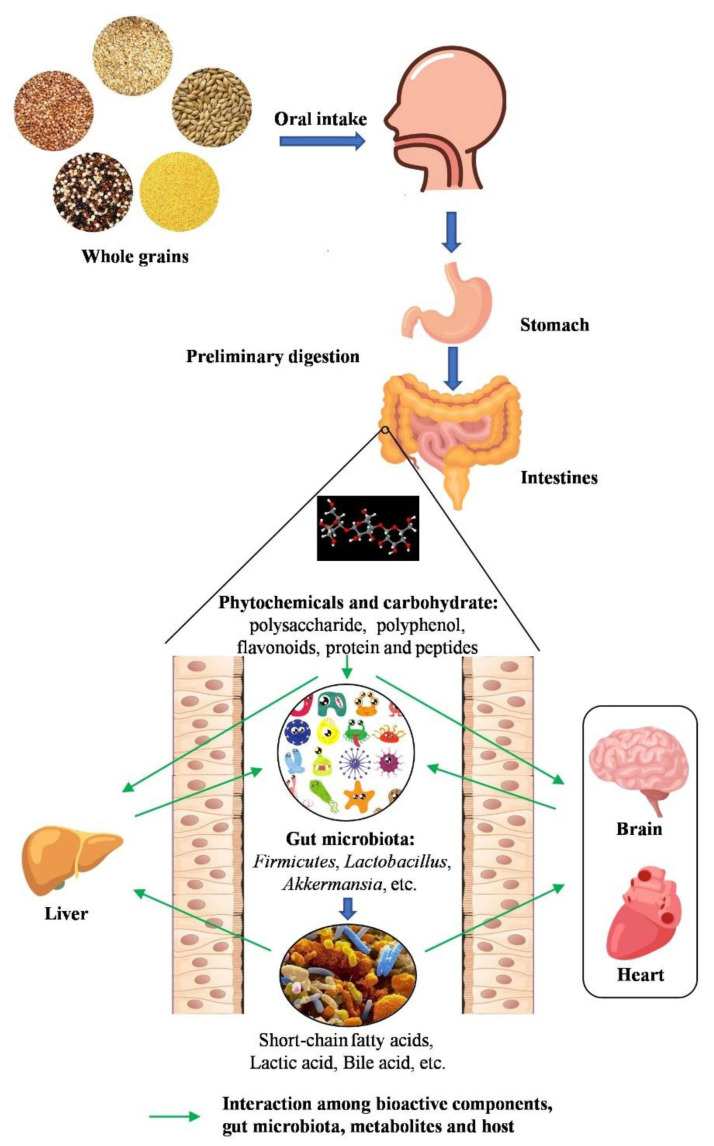
The interaction between whole grains and gut microbiota. The bioactive components of whole grains, which are produced after oral, gastric, and small intestine digestion, improve gut health by modulating gut microbiota. Phytochemicals and carbohydrates are metabolized by gut microbiota into short-chain fatty acids (SCFAs) and other small functional molecules. These functional components can regulate gut microbiota that contribute to exerting health effects on various diseases.

Although the non-digestible components in coarse grains are considered poor for gut microbial fermentation, the bioactive compounds of coarse grains have the potential to modulate the gut microbiota and thereby impact on consumer health [11]. David et al. suggested that increasing the proportion of coarse grains in the dietary structure is an effective way to promote a diversity of probiotics in the intestinal flora and alleviate a variety of chronic diseases [12]. A bifidogenic effect has been observed after intake of whole-grain cereals, and some *Bifidobacterium* strains have been reported as markers of a healthy gut microbiota [11,13]. In addition, oat, barley husks, and rye bran were reported to increase the growth of *Bifidobacteria* or *Lactobacillus*, which exert anti-tumor potential and enhance the formation of short-chain fatty acids (SCFAs) such as acetate and butyrate [14,15]. Butyrate is an important metabolite of gut microbial fermentation of carbohydrates due to its contribution to host colonic epithelial cell energy, its anti-inflammatory properties, and its anti-cancer effects [16]. Whole grains have been shown to decrease protein fermentation by the gut microbiota and may also increase the diversity of the microbiota [17,18]. Recently, the impact of food matrix components including fiber, protein, lipid oxidation products, and emulsions, and their effects on gut and metabolic health, has gained much attention. In this review, how the main components of coarse grains exert their beneficial health characteristics by regulating intestinal flora and the underlying mechanisms involved will be discussed (Figure 1).

## 2. Effects on Gastrointestinal Diseases

The gastrointestinal (GI) tract is the main interface for the interaction of dietary compounds and the organism. This complex system has developed multiple protective mechanisms against pathogens and toxic agents from the external environment, and it maintains a specialized balance of GI homeostasis, which is crucial for the digestive system to absorb nutrients and water [19]. However, poor lifestyle habits and imbalanced diet are the main reasons for the increasing incidence of gastrointestinal diseases such as inflammatory bowel disease (IBD), colorectal cancer (CRC), intestinal barrier dysfunction, etc.

Dietary interventions are not thought of as replacing agents in conventional medical therapies, but may be effective as complementary strategies for GI disorders [20]. Moreover, dietary molecules can influence the mechanisms of nutrient sensing and bioavailability, as well as the composition and metabolism of the gut microbiota, which ultimately might also affect GI homeostasis [21].

### 2.1. Inflammatory Bowel Disease

Recently, growing evidence points to the potential role of dietary bioactive compounds and microbiota modulation in IBD [22,23]. The effect of whole-grain components on regulating the gut microbiota is summarized in Table 1. For instance, intake of oat β-glucan significantly ameliorated the clinical features of ulcerative colitis in mice, attenuating the severe colonic inflammatory status and improving colonic mucosal barrier function concurrently with regulating gut-derived SCFAs and intestinal microbial metabolic profiles [24]. According to the authors, the reversed expression levels of zonula occludens-1 (ZO-1), occludin, claudin-1, and claudin-4 indicated that oat β-glucan promoted tight junction protein formation and restored intestinal epithelial barrier function. Moreover, they proved that pro-inflammatory cytokine interferon-γ (IFN-γ) and interleukin-6 (IL-6) were inhibited, while the production of SCFAs (especially acetate and propionate) increased, playing an important role in regulating intestinal epithelial tight junctions in UC mice. In a similar experiment, the low-molecular-weight form of β-glucan had a positive effect on the colon tissue of rats with lipopolysaccharide (LPS)-induced enteritis, resulting in changes in the levels of IL-10, IL-12, and tumor necrosis factor-α (TNF-α), as well as the number of intraepithelial lymphocytes (IELs) and lamina propria lymphocytes (LPLs) [25]. Moreover, a statistically significant reduction in the formation of hydroxybutyric acid and enhanced concentrations of lactic acid and propionic acid were observed, which was consistent with the number of lactic acid bacteria. This suggests an improvement in the alimentary tract contents.

Oat bran is one of the major byproducts in the processing of husked oat, and it contains relatively high levels of protein, minerals, vitamins, and soluble β-glucan [26]. He et al. investigated the effects of oat bran on nutrient digestibility, intestinal microbiota, and inflammatory responses in the hindgut of growing pigs. After supplementing with 10% oat bran (OB), the dietary nutrient digestibility showed no differences between the OB group and the control group on day 28. However, the mRNA expression of IL-8, nuclear factor-κB (NF-κB), and TNF-α was decreased in the OB group. Moreover, the abundances of *Prevotella*, *Butyricicoccus*, and *Catenibacterium* were increased in the colonic digesta, which may contribute to ameliorating inflammatory responses in the hindgut via enhancing the fermentation of fiber to produce SCFAs. Similarly, insoluble fiber in oat hulls could alleviate necrotic enteritis, and broilers fed with oat hulls had heavier gizzards compared with those without oat hulls. The gut microbiota composition was also improved by increasing the abundance of *Clostridium perfringens* and reducing the abundance of *Lactobacillus* and *Salmonellae* [27]. However, despite the fact that dietary fiber has been proved to be an effective strategy to prevent and relieve IBD through gut microbiota modulation, the function of soluble dietary fiber was found to be more favorable than that of insoluble dietary fiber. Tian et al. evaluated the bioactivity of insoluble dietary fiber from barley leaf (BLIDF) in inhibiting gut inflammation through regulating the intestinal microbiota in dextran sulfate sodium (DSS)-induced colitis mice. After intake of a BLIDF-supplemented diet for 28 days, they found that BLIDF markedly attenuated the severity of inflammation in DSS-induced acute colitis mice and increased the abundance of *Parasutterella*, *Erysipelatoclostridium*, and *Alistipes*, while the abundance of *Akkermansia* was significantly decreased. Moreover, BLIDF feeding can reverse the DSS-induced decline of short-chain fatty acids and secondary bile acids in mice feces [28]. Likewise, Li et al. explored the potential modulating roles of gut microbial metabolites of BL (barley leaf) in protecting against colitis and elucidated the underlying molecular mechanisms. They reported that supplementing with BL ameliorated DSS-induced gut microbiota dysbiosis and resulted in the enrichment of the microbiota-derived purine metabolite inosine, which could simulate peroxisome-proliferators-activated receptor γ (PPARγ) signaling to improve intestinal mucosal barrier functions in human colon epithelial cells [29].

It is also noteworthy that consumption of many purified dietary fibers has not been shown to increase the diversity of gut microbiota. The beneficial effects of whole grains on health are likely to be a combined result of many components within the grain rather than one specific component [30,31]. It was demonstrated that consumption of quinoa alleviated clinical symptoms according to the reduced disease activity index and the degree of histological damage. Compared to the control group, the species richness and diversity were increased in the quinoa consumption group, while abnormal expansion of the phylum *Proteobacteria* was decreased. Moreover, the overgrowth of the genera *Escherichia*/*Shigella* and *Peptoclostridium* was inhibited, whereas the abundances of *Firmicutes* and *Bacteroidetes* did not change significantly in the quinoa treatment group [32]. Another experiment investigated the effect of a buckwheat diet in a high-fat diet-induced gastritis mice model. The results showed that gastritis was lessened after intake of buckwheat, and the microbial dysbiosis induced by a high-salt diet could be recovered [33]. In the case of millet whole grain, its consumption by DSS-induced colitis murines alleviated the symptoms of enteritis to varying degrees, and completely alleviated DSS-induced dysbiosis. Moreover, the decreased levels of IL-6 and *claudin2* expression in the colon indicated that systemic inflammation and gut barrier function was improved after the consumption of millet whole grain [34].

### 2.2. Colorectal Cancer

Epidemiological studies have suggested that IBD patients exhibit a higher risk of CRC and that the incidence of cancer is positively correlated with the duration of IBD [35]. A recent study suggested that coarse cereal diets are beneficial for relieving CRC. Zhang et al. reported that intake of foxtail millet ameliorated AOM/DSS-induced colitis-associated CRC in mice via the activation of AHR and GPCRs and the inhibition of STAT3 phosphorylation by the microbial metabolites of the foxtail millet. In line with a previous study, the abundances of *Bifidobacterium* and *Bacteroidales_S24-7* were increased after millet consumption, compared to the control group [36]. Similarly, Yang et al. studied the effect of sorghum in an HFD-induced CRC mice model. The results showed that sorghum exhibited tremendous anti-CRC effects by suppressing the growth and metastasis of cancerous colon epithelial cells, as well as protecting against gut microbiota alterations linked to colitis [37].

Collectively, IBD, CRC, and intestinal barrier dysfunction could be alleviated by intake of a diet supplemented with coarse cereals or their bioactive components, due to the changes in relative gene expression, reduction of inflammatory cytokines (IL-6, IL-8, IL-10, etc.) and increased production of SCFAs demonstrated to be affected by the composition of gut microbiota.

**Table 1:** Effect of whole-grain components on gut microbiota of gastrointestinal diseases host.

Cereal	Component	Pathological Type	Study Characteristics	Pathological Parameters	Changes in Gut Microbiota	Reference
barley	leaf	colitis	female C57Bl/6J mice	IL-4 ↑, IL-10 ↑, TNF-α ↓, stool frequency ↑, gut transit time ↓, inosine ↑, guanosine ↑, glucose ↓, lactic acid ↑, body Weight ↑	*Proteobacteria* ↓, *Enterobacteriaceae* ↑, *Firmicutes* ↑, *Bacteroidetes* ↓, *Lactobacillus* ↑	[29]
	insoluble fiber	colitis	female C57BL/6J mice	IL-6 ↓, TNF-α ↓, IL-1β ↓, IL-4 ↓, IL-10 ↓, SCFAs ↑, secondary bile acids ↑, body Weight ↑	*Akkermansia* ↓, *Parasutterella* ↑, *Erysipelatoclostridium* ↑, *Alistipes* ↑, *Verrucomicrobia* ↓	[28]
buckwheat	whole grain	gastritis	SPF male C57BL/6 mice	IL-6 ↓, IL-1β ↓, IL-18 ↓, TH17 ↓, TGF-β ↓, IL-17A ↓, ILA ↑	*Unclassified_Lachnospiraceae* ↓, *Un-classified_Clostridiales* ↓, *Unclassified_Rikenellaceae* ↓, *Oscil-lospira* ↓, *Unclassified_S24-7* ↑, *Lactobacillus* ↑	[33]
millet	polyphenol	colorectal cancer	male C57BL/6J mice	COX-2 ↓and EMR1 ↓, PCNA cells ↓, caspase 3 ↑	*Firmicutes* ↑, *Bacteroidetes* ↓, *Prevotella* ↓, *Corprobacillus* ↑, *Parabacteroides* ↑, *AF12* ↑, *Coprococcus* ↑, *Oscillospira* ↑, *Ruminococcus* ↑, *Prevotella* ↓, *Desulfovibrio* ↓	[38]
	tryptophan and fiber	colorectal cancer	SPF male BALB/c mice	IL-6 ↓, IL-17 ↓, MPO ↓, MCP-1 ↓, serum C-P ↓, IFN-γ ↓, LSP ↓, COX-2 ↓, iNOS ↓, FOXP3 ↑, IL-22 ↑, ZO-1 ↑, IL-10 ↑, occludin ↑, Bcl-2 ↑, PCNA ↑, VEGF ↑, AHR ↑, SCFAs ↑	*Allobaculum* ↑, *Bifidobacterium* ↑, *Bacteroidales_S24-7* ↑, *Alistipes* ↓,	[36]
	whole grain	acute ulcerative colitis	male C57BL/6 mice	IL-6 ↓, claudin2 ↓, ZO-1 ↑, occludin ↑, claudin 2↓	*Muribaculaceae* ↑	[34]
oat	bran	inflammatory	pig	IL-8 ↓, colonic IL-8 ↓, NF-κB ↓, TNF-α ↓	*Catenibacterium* ↑, *Peptococcus* ↓, *Prevotella* ↑, *Butyricicoccus* ↑, *Catenibacterium* ↑, *Coprococcus* ↓, *Desulfovibrio* ↓	[39]
	β-glucan	enteritis	male SD rats	IL-12 ↓, IL-1α ↓, β, IL-6 ↓, IL-10 ↑, TNF-alpha ↑, lactic acid ↑, propionic acid ↑, hydroxybutyric acid ↓	-	[25]
	fiber	necrotic enteritis	broiler chickens	succinic acid ↑, acetic acid ↓, propionic acid ↓, valeric acid ↓	*Perfringens* ↑, *Lactobacillus* ↓, *Salmonellae* ↓,	[27]
quinoa	whole grain	colonic colitis	male C57BL/6 mice	IL-6 ↓, IL-1β ↓, IFN-γ ↑	*Proteobacteria* ↓, *Escherichia/Shigella* ↓, *Peptoclostridium*↓, *Bacteroidetes* ↑, *Verrucomicrobia* ↑	[32]
sorghum	whole grain	colorectal cancer	humanfemale C57BL/6 mice	-	*Bacteroides* ↑, *Fusobacterium* ↑, *Dorea*, *Porphyromonas* ↑, *Pseudomonas* ↓, *Prevotella* ↓, *Acinetobacter* ↓, *Catenibacterium* ↓	[37]

“↑” and “↓” indicated the “increase” and the “decrease” of content or expression.

## 3. Effects on Cardiovascular Diseases

Hyperlipidemia is the main cause of cardiovascular diseases such as hypertension, atherosclerosis, stroke, etc. [40]. Hyperlipidemia is a chronic lipid disorder that causes abnormally increased levels of total cholesterol (TC), triglycerides (TG), and low-density lipoprotein cholesterol (LDL-C), and a decreased level of high-density lipoprotein cholesterol (HDL-C) [41]. Although hyperlipidemia can be controlled using pharmacological strategies, long-term exposure to drugs may lead to unpleasant side effects [42]. Therefore, natural lipid-lowering drugs without adverse effects have been considered the best alternatives against hyperlipidemia. Increasing evidence has indicated that the gut microbiota, which is involved in lipid absorption and liver cholesterol metabolism, is closely associated with hyperlipidemia [43,44].

### 3.1. Hyperlipidemia

Recent studies have proved that the consumption of some beneficial whole-grain products is associated with a lower risk of hyperlipidemia and has a close relationship with alterations in the gut microbiota [45]. The composition, content, and functional properties of phytochemicals in grains, including flavonoids, polysaccharides, polyphenols, and polypeptides, have been shown to possess a potential antihyperlipidemic effect by ameliorating lipid profiles and gut dysbiosis in vivo [46]. Here, we present a summary of the bioactive substances of whole grains in the regulation of gut microbiota (Table 2). In a high-fat diet (HFD)-induced hyperlipidemia mice model, flavonoids from whole-grain oat (FO) improved serum lipid profiles and decreased lipid deposition. RT-qPCR and Western blot analysis showed that FO suppressed lipogenesis and promoted lipolysis, BA synthesis, and efflux to feces through the FXR pathway. Moreover, 16s rRNA sequencing revealed that FO significantly increased *Akkermansia* and significantly decreased *Lachnoclostridium*, *Blautia*, *Colidextribacter*, and *Desulfovibrio*, which was correlated with hyperlipidemia-related parameters. Thus, FO exhibited hyperlipidemic inhibitory activity by regulating the gut–liver axis (BA metabolism and gut microbiota) [47]. Likewise, intake of embryo-remaining oat rice (EROR) dramatically improved the lipid profile in the serum and liver. Furthermore, EROR supplementation significantly increased the total SCFAs, acetate, and propionate and promoted the abundance of SCFA-producing bacteria. In addition, EROR consumption caused an alteration in the composition of the indigenous flora, with increases in *Bifidobacterium* and *Akkermansia* and decreases in *Rombutsia*, *Fusicatenibacter*, *Holdemanella*, and *Turicibacter* that were correlated with the lipid-metabolism-related indices [48].

Plant-derived polysaccharides are natural polysaccharides and are non-toxic with few side effects [49]. Cao et al. found that oral administration of quinoa polysaccharide mitigated dyslipidemia and reduced hepatic lipid accumulation by decreasing the contents of TG, LDL-C, glutamic oxaloacetic transaminase (AST), glutamic pyruvic transaminase (ALT), and malondialdehyde (MDA). Concurrently, the species richness and gut microbiota community structure were positively regulated by intake of quinoa polysaccharide. Furthermore, the results of 16s rRNA sequencing showed that the ratio of *Firmicutes* and *Bacteroides* (F/B ratio) and the abundance of *Proteobacteria*, *Desulfovibrio*, and *Allobaculum* were decreased, which was beneficial in mitigating intestinal inflammation and ameliorating the serum lipid levels. Thus, quinoa polysaccharide could reduce the numbers of potentially pathogenic bacteria to decrease infection, inflammation, and hyperlipidemia [50].

Compared to most cereals, the protein fractions of quinoa and buckwheat have high nutritive value. In the research of Fotschki and co-workers, the effects of quinoa and buckwheat protein-rich flours on the growth parameters, intestinal microbial activity, and plasma lipid profile were studied. The studied flours favorably reduced the plasma total cholesterol and LDL cholesterol, increased the levels of plasma IL-6 and alanine transaminase, and regulated the microbial production of SCFAs in rats [51]. Among these SCFAs, butyric acid was significantly elevated. This is the preferred energy source for colon epithelial cells and could decrease the pH together with other SCFAs [52]. Moreover, butyric acid is able to activate AhR and negatively regulates several enzymes, including fatty-acid synthase and a cholesterol metabolism regulator in the form of sterol regulatory element-binding protein-1c [53]. In a similar experiment, male C57BL/6 mice were fed an HFD with buckwheat protein (BWP) to evaluate the effect of BWP in attenuating dyslipidemia. The levels of TC, TG, LPS, TNF-α, and IL-6 in the mice fed on the HFD with BWP were significantly lower than those on an HFD with casein. In addition, BWP markedly increased the total bile acids and short-chain fatty acids, and also improved the abundances of *Lactobacillus*, *Bifidobacterium*, and *Enterococcus*, and decreased the abundance of *Escherichia coli* in HFD-fed mice. Notably, the *Bifidobacterium* population was closely related to the plasma lipids content [54]. A previous study reported that the presence of specific prebiotics in buckwheat may stimulate the growth of probiotics and other beneficial gut bacteria present in the colon and help to maintain healthy gut conditions [55]. Zhou et al. explored the effect of buckwheat fermented milk on the intestinal flora and SCFAs of HFD-fed rats. The buckwheat fermented milk remarkably repressed the increases in LPS levels in the colon and the antioxidant indexes in HFD-fed rats. In addition, buckwheat fermented milk significantly enhanced the abundance of the genus *Akkermansia* and decreased the abundance of the *Lachnospiraceae* family [56]. It has been proved that the species belonging to *Lachnospiraceae* are linked to the development of obesity and type 2 diabetes in ob/ob rats, whereas *Akkermansia*, belonging to *Verrucomicrobia*, plays an important role in treating obesity, inflammation, type 2 diabetes, and metabolic syndrome [57,58].

There has been growing evidence suggesting that intake of oat and buckwheat is associated with a reduction in serum cholesterol [59,60]. Zhou and co-workers reported that whole-grain oat (WGO) flour improved insulin sensitivity and the plasma cholesterol profile compared with low-bran oat (LBO) flour, resulting in lower plasma insulin, C-peptide, total cholesterol, and non-HDL cholesterol, associated with the changes in cecal microbiota composition. The relative abundances of *Prevotellaceae*, *Lactobacillaceae*, and *Alcaligenaceae* families were increased in the WGO group, while abundances of *Clostridiaceae* and *Lachnospiraceae* families were greater in the LBO group [61]. Subsequent analysis investigated the cholesterol-lowering effect of a whole-grain oat-based breakfast in a cardio-metabolic “at risk” population. It was observed that TC and LDL-C were significantly decreased after WGO consumption compared to the control group. Furthermore, a significant change was also observed in the relative abundances of fecal *bifidobacteria*, *lactobacilli*, and total bacterial count, which were all increased after WGO supplementation [62]. In another similar study, the effect of oat- and tartary-buckwheat-based food (OF) on cholesterol reduction and the gut microbiota in hypercholesterolemic hamsters was evaluated. After intake of OF for 30 days, the plasma TC and LDL-C, liver TC, cholesterol ester (CE), and TG were significantly decreased, whereas the concentrations of acetate, propionate, butyrate, and total SCFAs were significantly increased, in the hamsters fed with OF. Moreover, 16s rRNA sequencing results showed that the relative abundances of *Erysipelotrichaceae*, *Ruminococcaceae*, and *Lachnospiraceae* were decreased and the relative abundance of *Eubacteriaceae* was increased, beneficially influencing the fatty acid metabolism [60]. These results indicated that the increased SCFAs and the improved gut microbiota served their hypocholesterolemic function.

### 3.2. Hypertension

Hypertension is a major risk factor for cardiovascular disease [63]. Dietary approaches to stop hypertension (DASH) involve diets rich in fruits, vegetables, whole grains, and low-fat dairy products, with a reduced content of saturated and total fat, and are recommended for adults with elevated hypertension or blood pressure [64]. In a randomized controlled trial, the participants who consumed oat bran (30 g/d) had a lower office systolic blood pressure and office diastolic blood pressure, and the use of antihypertensive drugs in the DF group was reduced. Moreover, the abundances of *Bifidobacterium* and *Spirillum* were significantly elevated [65]. Similarly, the BP-lowering effect of buckwheat iminosugar d-fagomine was measured in sucrose-induced hypertensive rats. D-fagomine reduced sucrose-induced hypertension, urine uric acid, and steatosis, without impairing glucose tolerance and without affecting perigonadal fat deposition. The intestinal microbiota was also influenced under a high-sucrose (HS) diet with D-fagomine (FG): the *Bacteroidetes*/*Firmicutes* ratio was slightly increased compared to the HS diet group, which was consistent with the changes among the standard, HS, and HS+FG groups. Furthermore, the increased numbers of excreted *Enterobacteriales* in the HS+FG group proved the inhibitory activity of d-fagomine on epithelial adhesion of *Escherichia coli* [66]. Recently, Guo et al. studied the antihypertensive effect of quinoa protein on spontaneously hypertensive rats (SHRs). During the five weeks of intake of quinoa protein, the blood pressure (BP) was significantly decreased, while the alpha diversity was increased, and the microbial structure was changed. Subsequent analysis revealed that the abundances of *Turicibacter* and *Allobaculum* genera were negatively correlated with BP [67].

### 3.3. Nonalcoholic Fatty Liver Disease

In general, patients with dyslipidemia also suffer from nonalcoholic fatty liver disease (NAFLD), and clinical manifestations include excessive accumulation of lipids, including TG and FAs, in hepatocytes [68]. Previous studies suggested that the gut microbiome is involved in the development of dyslipidemia and NAFLD [69], whereas the intestine and liver are intrinsically linked and strongly interdependent in metabolic functions [70,71]. For example, common buckwheat consumption significantly improved physiological indexes and biochemical parameters related to dyslipidemia and NAFLD in mice fed with an HFD. In addition, the fecal bile acid (BA) abundance was increased and fecal SCFA reductions were recovered after intake of buckwheat. Moreover, it has been found that buckwheat intervention is more beneficial than simvastatin (10 mg/kg/d) in ameliorating the dysbiosis of intestinal microbial populations induced by an HFD. The abundances of *Lactobacillus*, *Blautia*, and *Akkermansia* were markedly increased, whereas the abundances of *Allobaculum*, *Desulfovibrio*, and *Bacteroidales_S24-7* were decreased under buckwheat intervention [71]. It has been reported that the possible mechanism of the lipid-lowering effect of *Lactobacillus* may be to produce lactic acid through intestinal glucose metabolism and promote blood lipid metabolism γ-production of aminobutyric acid and other useful substances [72]. The increase in the genus *Blautia* might occur mainly because of the syntrophic effect [18]. *Akkermansia* could consume host-derived mucins such as carbon and nitrogen sources and ferment indigestible carbohydrates, while mainly producing propionic acid. In contrast, *Allobaculum* showed a strong positive association with lipid metabolic phenotypes [73]. These changes were consistent with previous studies. However, the abundance of *Bacteroidales_S24-7*, reported to be involved in host–microbe interactions that impact gut function and health [74], was considerably increased by the HFD supplement but reduced by the buckwheat intervention. Recent studies have shown that *Monascus* fermentation can increase the content of polyphenols or flavonoids in the fermentation substrate [75]. Huang and co-workers found that oral administration of *Monascus purpureus*-fermented common buckwheat (HQ) significantly inhibited the abnormal growth of body weight and epididymal white adipose tissue (eWAT), prevented the hypertrophy of epididymal adipocytes, and alleviated some biochemical parameters of serum and liver related to lipid metabolism in HFD-induced mice. Histological analysis also exhibited that HQ supplementation reduced the excessive accumulation of lipid droplets in the livers. Furthermore, HQ consumption significantly changed the structure of the intestinal microflora. In the comparison of the HFD and HQ groups, intake of HQ markedly increased the relative levels of *Ruminiclostridium*, *Lacobacillus*, and *Alistipes* and decreased the abundances of the *Bacteroidales S24-7* group and *Clostridiales XIII* in HFD-induced mice. Moreover, UPLC-QTOF/MS-based liver metabolomics showed that HQ consumption exerted remarkable effects on the metabolic pathways of primary bile acid biosynthesis, ether lipid metabolism, glycine, serine and threonine metabolism, glutathione metabolism, pyrimidine metabolism, amino sugar and nucleotide sugar metabolism, etc. These results demonstrated that NAFLD could be attenuated by HQ intervention through regulating the hepatic metabolite profile and gut microbiota. [76].

### 3.4. Atherosclerosis

Atherosclerosis, the major cause of cardiovascular disease, is a chronic inflammatory disease, which can lead to vascular cognitive impairment and dementia via gradual thickening of large artery walls, reducing blood flow [77,78]. In atherosclerosis, inflammation can damage vascular endothelial cells and promote the formation of foam cells, causing lesions. Increased inflammation and lipid accumulation play key roles in the development of atherosclerosis [79,80]. In the classic atherosclerosis model, the anti-atherosclerotic activity of millet shell polyphenols (MSPs) was measured in ApoE-/-mice fed with a high-fat diet. The results showed that the atherosclerotic plaques in the aorta were effectively inhibited by MSPs, and the LPS and inflammatory cytokine levels, including TNF-α and IL-1β, were also decreased. Conversely, the mRNA expression of tight junction proteins such as occludin, zona occludens-1, and claudin1 was significantly increased in the MSPs group. Moreover, the structure of gut microbiota in ApoE-/-mice with a high-fat diet was improved under consumption of MSPs, resulting in increased abundances of *Oscillospira* and *Ruminococcus* and a decreased abundance of *Allobaculum* at the genus level [81]. Recently, the LDL receptor knock-out mice (LDLR-/-) model, along with a high LDL-cholesterol concentration, has been the established model for atherosclerotic-related cognitive dysfunction [82]. Gao et al. evaluated the effects of an oat-fiber intervention on cognitive behavior by targeting the neuroinflammation signal and the microbiome–gut–brain axis in a mouse model of atherosclerosis. They found that dietary oat fiber retarded the progression of cognitive impairment in a mouse model of atherosclerosis, due to the effect of its metabolites and SCFAs on the diversity and abundance of the gut microbiota that produced anti-inflammatory metabolites, leading to repressed neuroinflammation and reduced gut permeability through the microbiome–gut–brain axis [83].

Overall, increasing numbers of bioactive components in coarse cereals have been proved to possess a hypolipidemic effect, leading to decreased TC, TG, HDL-C, LDL-C, AST, ALT, and MDA. The key genes of lipid metabolism and inflammatory factors are inhibited, whereas the composition of gut microbiota may have an important role in hyperlipidemic regulation. Among these changed bacteria, the F/B ratio, *Akkermansia, Prevotellaceae*, and *Lactobacillaceae* were thought to contribute to regulating serum lipid, while the abundances of *Allobaculum, Lachnospiraceae*, and *Bacteroidales S24-7* were inversely correlated with levels of plasma lipids.

**Table 2:** Effect of whole-grain components on gut microbiota of cardiovascular diseases host.

Cereal	Component	Pathological Type	Study Characteristics	Pathological Parameters	Changes in Gut Microbiota	Reference
buckwheat	fermented milk	dyslipidemia	males C57BL/6 rats	weight ↓, LPS ↑, TNF-α ↑, IL-6 ↑, SOD ↓, T-AOC ↓, CAT ↓, MAD ↑, acetic ↑, propionate ↑, total acid ↑	*Firmicutes* ↓, *Bacteroidetes* ↑, *norank-f-Bacteroidales-S24-7-group* ↓, *unclassified-f-Lachnospiraceae* ↓, *Blautia* ↓, *Enterobacter* ↑, *Akkermansia* ↑	[56]
	whole-grain-based food	nonalcoholic fatty liver disease	male Kunming mice	SCFAs ↑, bile acid ↑, weight ↓, TC ↓, TG ↓, LDL-C ↓, LPS ↓, ALT ↓, AST ↓	*Blautia* ↑, *Lactobacillus* ↑, *Streptococcus* ↑, *unclassified_Rikenellacea* ↑, *Desulfovibrio* ↓, *Bacteroidales_S24-7* ↓, *Allobaculum* ↓	[71]
		hypercholesterolemia	male gold hamsters	TC ↓, LDL-cholesterol ↓, CE ↓, TG ↓, BA ↑, acetate ↑, propionate ↑, butyrate ↑, SCFAs ↑	*Erysipelotrichaceae* ↓, *Ruminococcaceae* ↓, *Lactobac-illaceae* ↓, *Lachnospiraceae* ↓, *Eubacteriaceae* ↑, *Bacteroide-tes* ↓, *Ruminococcus-1* ↓, *Ruminococcus-2* ↓, *Ru-minococcuceae-UGG-014* ↓	[60]
	fermented common buckwheat	non-alcoholic fatty liver disease	SPF male Kunming mice	weight ↓, TG ↓, TC ↓, LDL-C ↓, AST ↓, ALT ↓, TBA ↓, NEFA ↓, HDL-C ↑	*Ruminiclostridium* ↑, *Lacobacillus*, *Alistipes* ↑, *Bacteroidales S24-7 group* ↓, *Clostridiales XIII* ↓	[76]
	fermented black tartary buckwheat	hyperlipidemia	male SD rats	tyrosine ↑, lysine ↑, total flavonoids ↑, total polyphenols ↑, quercetin ↑, kaempferol ↑, rutin ↓	*Lactobacillus* ↑, *Faecalibaculum* ↑, *Allobaculum* ↑, *Romboutsia* ↓	[84]
	d-fagomine	hypertension	male WKY rats	uric acid ↑	*Bacteroidetes* ↑, *enterobacteriales* ↑, *E. colipopulations* ↑	[66]
	protein	dyslipidemia	SPF Male C57BL/6 mice	TC ↓, TG ↓, total bile acids ↑, SCFAs ↑, LPS ↓, TNF-α ↓, IL-6 ↓	*Lactobacillus* ↑, *Bifidobacterium* ↑, *Enterococcus* ↑, *Escherichia coli* ↓	[54]
millet	arabinoxylan	lipid derangements, endotoxemia	male mice (Swiss albino, LACA strain)	total cholesterol ↓, HDL-C ↓, LDL-C ↓, VLDL-C ↓, NEFA ↓, FASN ↓, ACC ↓, SREBP-1c ↓, HMGCOA-R ↓, HLST ↑, TNF-α ↓, IL-1β ↓, IL-6 ↓, CRP ↓	*Bacteroidales S24-7* ↑, *Blautia sp.* ↓, *Lactobacillus sp.* ↓, *Bifidobacterium sp.* ↓, *Roseburia* ↓, *Bacteroidetes* ↓	[85]
	millet shell polyphenols	atherosclerosis	male ApoE−/−mice	LSP ↓, TNF-α ↓, IL-1β ↓, occludin ↑, zona occludens-1 ↑, claudin1 ↑	*Verrucomicrobia* ↓, *Actinobacteria* ↓, *Oscillospira* ↓, *Ruminococcus* ↓, *Bacteroidetes* ↑, *Allobaculum* ↑	[81]
	vitexin	brain oxidative stress and inflammation	male C57BL/6 N mice	SOD ↑, CAT ↑, GPx ↑, MDA ↓, Keap1 ↓, TNF-α ↓, IL-1β ↓, IL-6 ↓, IL-10 ↓	*Firmicutes* ↓, *Verrucomicrobiota* ↑, *Lachnospiraceae* ↑, *Escherichia-shigella* ↑	[86]
oat	fiber	hypertension	human	SCFAs ↑	*Spirillum* ↑, *Bifidobacterium* ↑, *Trichosporium* ↑	[65]
	fiber	atherosclerosis	male LDLR−/−mice	GFAP ↓, IBα1 ↓, SCFAs ↑, zonula occludens-1 ↑, occludin ↑, LSP ↓	*Actinobacteria* ↑, *Peptostreptococcaceae* ↑, *Coriobacteriaceae* ↑, *Eisenbergiella* ↑, *Romboutsia* ↑, *Rikenellaceae* ↓, *Anaerotruncus* ↓, *Parabacteroides* ↓	[83]
	flavonoids	hyperlipidemia	male C57BL/6N mice	weight ↓, PPARα ↑, CPT-1 ↑, CYP7A1 ↑, FXR ↑, TGR5 ↑, NTCP ↑, BSTP ↑, SREBP-1c ↓, FAS ↓, ASBT ↓	*Akkermansia* ↑, *Lachnoclostridium* ↓, *Blautia* ↓, *Colidextribacter* ↓, *Desulfovibrio* ↓	[47]
	β-glucan	cardiovascular disease	apo-E−/−mice	total cholesterol ↓	*Verrucomicrobia* ↓, *Proteobacteria* ↑, *Peptostreptococcaceae* ↓, *Christenellaceae* ↓, *Desulfovibrionaceae* ↑, *Bacteroidaceae* ↑, *Helicobacter* ↑, *Akkermansia* ↑, *Ruminococcus spp.* ↑	[87]
	whole grain	cholesterol	human	TC ↓, LDL-C ↓	*Bifidobacteria* ↑, *lactobacilli* ↑	[62]
	whole grain	cholesterol and diabetes	male C57BL/6J mice	weight ↓, insulin ↓, plasma total cholesterol ↓	*Lachnospiraceae* ↑	[61]
	oat ethanol extracts	lipid metabolic disorder	male SD rats	the total SCFAs ↑, acetate ↑, propionate ↑, SREBP-1C ↓, FAS ↓, HMGCR ↓, TG ↑, TC ↑, LDL-C ↑, HDL-C ↑	*Bifidobacterium* ↑, *Akkermansia* ↑, *Rombutsia* ↓, *Fusicatenibacter* ↓, *Holdemanella* ↓, *Turicibacter* ↓	[48]
quinoa	soluble polysaccharide	hyperlipidemia	male SD rats	TG ↓, LDL-C ↓, MDA ↓, ASTl ↓, TC ↓, ALT ↓, HDL-C ↑, SCAFs ↑	*the ratio of Firmicutes* ↓, *Bacteroides* ↓, *Proteobacteria* ↓, *Desulfovibrio* ↓, *Allobaculum* ↓	[50]
	protein	antihypertensive	SHRs and WKY rats		*Allobaculum* ↑, *Turicibacter* ↑, *Staphylococcus* ↑	[67]

“↑” and “↓” indicated the “increase” and the “decrease” of content or expression.

## 4. Effects on Obesity

Obesity has become a serious public health problem all over the world [88], and is closely associated with several diseases including hypertension, type 2 diabetes, coronary artery disease, chronic inflammation, and cancer [89,90,91]. Previous studies have proved that the occurrence and development of obesity is related to changes in the composition and metabolism of the gut microbiota [92]. In the above section, coarse cereals and their bioactive substances exhibited lipid-lowering efficacy in cardiovascular disease. Furthermore, they also have potential for the management of obesity and several related metabolic disorders. In an experiment by Ji et al., dietary intake of a mixture of coarse cereals could prevent body weight gain and fat accumulation, improve blood glucose tolerance and serum lipids levels, and reduce systemic inflammation in HFD-fed mice. Moreover, the relative abundances of *Lactobacillus* and *Bifidobacterium* could be increased after supplementing with a mixture of coarse cereals, which might contribute to the anti-obesity activity [93].

In addition to mixed coarse cereals, many single grains have also been proved to promote weight reduction. For instance, obese db/db mice were fed commercial diets with and without quinoa supplementation for eight weeks. The results showed that the quinoa-supplemented diet delayed the body weight gain of mice. Moreover, 16s sequencing analysis showed that the relative abundances of *Enterococcus*, *Turicibacter*, *Akkermansia*, and *Bacteroidetes* returned to normal levels as shown in the lean group [94]. Among these groups, *Turicibacter* was considered to be a group with several beneficial effects, whereas *Akkermansia* was a mucin-degrading bacterial taxon that was negatively correlated with beneficial body weight effects [95,96,97]. An opposite result was observed in this study: the abundance of *Firmicutes* was higher in lean mice compared to db/db mice, which was mainly related to their diets [94]. Likewise, the effect of barley supplementation on the fecal microbiota was also measured in obese db/db mice. However, the fecal microbiota results showed that barley consumption contributed to a phylogenetically unique microbiota distinct from both obese and lean controls. Moreover, the content of cecal butyrate was similar in obese mice, whereas succinic acid was lower in the barley group. Compared to the obese group, barley supplementation was also relevant, with lower plasma insulin and resistin levels. [98]. Furthermore, previous studies reported higher and lower levels of cecal butyrate during intake of whole wheat and quinoa, respectively, compared to obese controls [94,99].

Along the same lines, the effect of a whole barley (WB) supplement on preventing obesity was evaluated in germ-free (GF) C57BL/6J mice and human-flora-associated (HFA) mice. The results showed that WB prevented obesity and hypercholesterolemia in the GF and HFA mice. The postulated mechanism was the inhibition of cholesterol synthesis due to the downregulation of 3-hydroxy-3-methylglutaryl coenzyme A reductase and sterol regulatory element-binding protein-1c, thereby decreasing cholesterol accumulation. In addition, intake of WB enriched a variety of bacterial genera that are negatively associated with obesity, including *Bacteroides*, *Parabacteroides*, and *Clostridium* cluster XIVa, suggesting that WB counteracted gut dysbiosis in obese mice [100]. In an experiment by Zhong et al., whole-grain barley and barley malt consumption improved the structure of cecal microbiota in HFD-induced rats. Compared to the control group, the barley group had lower abundances of *Firmicutes* and *Deferribacteres* and higher abundances of *Verrucomicrobia* and *Actinobacteria*. At the genus level, the abundances of *Akkermansia*, *Ruminococcus*, *Blautia*, and *Bilophila* were increased, while *Turicibacter* and *Roseburia* were more abundant in the malt group. Most genera correlated with acetic and propionic acids. However, *Roseburia* and *Turicibacter* correlated with butyric acid, and Clostridium and *Akkermansia* correlated with succinic acid [101]. It has also been demonstrated that a synbiotic consisting of *Lactobacillus plantarum* S58 (LP.S58) and hull-less barley β-glucan (β-G) attenuates lipid accumulation in HFD-induced mice. In addition, LP.S58 and β-G alleviated gut microbiological dysbiosis in HFD-fed mice. The abundances of *Helicobacter*, *Ruminococcaceae*, and *Bacteroides* were markedly reduced after synergistic treatment with LP.S58 and β-G, while the abundances of *Lactobacillus*, *Allobaculum*, *Turicibacter*, *Dubosiella*, *Akkermansia*, and *Faecalibaculum* were increased [102].

Likewise, Hereu et al. investigated the effect of combined d-fagomine and ω-3 polyunsaturated fatty acids (ω-3 PUFAs) on weight gain and beneficial gut bacterial strains. D-fagomine was first isolated from the seeds of buckwheat and could reduce weight gain in the rats fed with ω-3 PUFAs, compared to the controls. Moreover, intake of ω-3 PUFAs promoted the diversity of the gut microbiota by increasing the abundance of *Bacteroidetes* in healthy rats and mitigated the age-related reduction of *Lactobacillus* and *Bifidobacterium*, which are presumed to be beneficial [103,104]. Eicosapentaenoic acid (EPA, 20:5, n-3) and docosahexaenoic acid (DHA, 22:6, n-3) are the major ω-3 PUFAs, and they are considered to be crucial components in preventing cardiovascular diseases [105]. Previous studies reported that EPA and DHA significantly increased the abundances of *Firmicutes* (*Lactobacillus* genus) and *Bifidobacteria* in mice fed an HFD [106,107]. According to the authors, the d-fagomine and ω-3 PUFAs combination group gained less weight compared to the controls and the ω-3 PUFAs group, and the combination helped maintain the relative populations of *Bacteroides* and *Prevotella* in the rat intestinal tract, providing the anti-inflammatory and cardiovascular benefits of ω-3 PUFAs [108]. It is well known that tartary buckwheat is rich in flavonoids, and dietary rutin or quercetin in buckwheat can ameliorate lipid metabolism, whereas buckwheat-resistant starch may also have beneficial effects on lipid metabolism. For example, Peng et al. proved that quercetin could significantly reduce body weight, serum TC, LDL-C, insulin, and TNF-α, while buckwheat significantly increased the weight of the rats. Moreover, rutin, quercetin, and buckwheat tended to decrease fat deposition in the liver of rats but had little effect on production of SCFAs. It has also been found that they can shape the specific structure of the gut microbiota [109]. Zhou et al. investigated the regulatory effects of tartary-buckwheat-resistant starch (BRS) on blood lipid levels and the gut microbiota in HFD-induced mice. After treatment, plasma levels of TC, TG, glucose, and cytokines were markedly decreased and the antioxidant capacity in the liver and duodenum was enhanced. Furthermore, the gut microbiota composition was regulated after the intake of BRS, and the populations of *Bacteroides*, *Blautia*, *Lactobacillus*, *Bifidobacterium*, and *Enterococcus* were promoted, while the growth of *Faecalibaculum*, *Erysipelatoclostridium*, and *Escherichia coli* was inhibited [110,111].

Like quinoa, millet lacks gluten and is rich in dietary fiber, polyphenols, and minerals, which means that it could be explored as a healthy food. Recently, Sarma and co-workers investigated the protective role of whole-grain kodo millet supplementation in HFD-induced mice. They found that millet consumption ameliorated glucose tolerance and inhibited increases in the serum cholesterol and lipid parameters but did not influence weight gain. Moreover, millet increased the beneficial gut bacterial abundances, including *Lactobacillus spp.*, *Bifidobacteria*, *Akkermansia*, and *Roseburia spp.* [112].

Grain sorghum is the world’s fifth most produced cereal crop, but it is commonly grown only in specific areas. Sorghum contains various types of polyphenols, which are found in the bran [113]. Ashley et al. studied the effect of grain sorghum polyphenols on the gut microbiota of different weight classes. There were no significant changes in total production of SCFAs between normal-weight and overweight/obese groups. In contrast, a combination of sorghum bran polyphenols and fructo-oligosaccharides could increase the populations of *Bifidobacterium* and *Lactobacillus*, and sorghum bran polyphenols independently enhanced the abundances of *Roseburia* and *Prevotella* [114].

The same clinical parameters were observed in obesity as in cardiovascular diseases. There were some differences in the flora changes, such as for *Allobaculum* and *Blautia*, which were increased, but further study is needed on role of the gut microbiota in the regulation of obesity.

## 5. Effects on Diabetes

Diabetes and its related diseases have reached alarming levels worldwide [115]. Type 2 diabetes (T2D) accounts for the vast majority of cases of diabetes. Unhealthy dietary habits, including excess consumption of sugar, oil, and flour, leads to obesity, which is the main cause of T2D [116]. Growing evidence suggests that T2D can be prevented and treated through intake of whole grains [117]. However, there are few studies about the hypoglycemic effect of grains via regulation of the gut microbiota. Recently, Ren et al. evaluated the hypoglycemic effect of foxtail millet (FM) in high-fat-diet and streptozotocin-induced diabetic rats, and the results exhibited remarkably decreased fasting glucose (FG), glycated serum protein, and areas under the glucose tolerance test. A 16s rRNA sequencing analysis showed that FM consumption significantly increased the relative abundances of *Lactobacillus* and *Ruminococcus_2*, which were significantly negatively correlated with FG and 2 h glucose. Subsequently, a Spearman’s correlation analysis indicated a complicated set of interdependences among the gut microbiota and metabolic parameters [118].

Regarding the protein of FM, the hypoglycemic impact of different protein types was investigated by Fu and co-workers. Protein isolates from raw and cooked FM improved glucose intolerance and insulin resistance in diabetic mice. However, only the protein of cooked FM recovered the weight loss trend and alleviated lipid disorders in diabetic mice. The results of the gut flora showed that intake of FM protein isolates simulated the population of *Akkermansia* and decreased the abundance of *Lachnospiraceae* [119]. *Akkermansia* was reported to be negatively associated with obesity, insulin resistance, diabetes, and other metabolic diseases [120]. In this study, the increased population of *Akkermansia* may be attributed to the fact that the amino acids produced by the hydrolysis of millet protein isolate in the intestine can be metabolized by the intestinal flora, finally producing amines and polyamines through a decarboxylation reaction, which was demonstrated to improve the *Akkermansia* abundance [121,122]. Moreover, the protein isolated from the raw foxtail millet increased the abundances of the *Clostridiaceae_1* family and *Faecalibaculum* genus, whereas protein isolate from the cooked foxtail millet increased the level of the genus *Ruminococcaceae_UCG-014*. A previous study proved that the populations of the *Clostridiaceae_1* family and *Ruminococcaceae_UCG-014* genus were negatively associated with the circulating isoleucine level in plasma [123]. Isoleucine belongs to the branched-chain amino acids (BCAAs) and has been shown to be positively correlated with the development of obesity and insulin resistance [124]. Furthermore, *Faecalibaculum* was found to produce lactic acid, and its metabolites were reported to have anti-diabetic effects [125]. More recently, these authors investigated the hypoglycemic effect of prolamin from cooked foxtail millet (PCFM) on T2DM mice. PCFM ameliorated glucose metabolism disorders associated with type 2 diabetes, and diabetes-related and gut microbiota dysbiosis in mice. PCFM inhibited the growth of *Lachnospiraceae_UCG-006*, *Anaerotruncus*, and *Butyricimonas*, as well as promoting the populations of *Odoribacter*, *Blautia*, and *Akkermansia* [126]. *Lachnospiraceae_UCG-006* was revealed to be positively correlated with TG, TC, HDL-C, and LDL-C [127]. Moreover, *Anaerotruncus* genus was reported to be positively related to glucose intolerance in HFD mice, whereas a positive correlation between the abundance of *Butyricimonas* and fasting blood glucose in T2DM patients has been observed [128,129]. In contrast, *Odoribacter* was negatively related to steady-state plasma glucose in pre-diabetic individuals [130]. Moreover, the gut microbiota composition was markedly altered and glucose metabolism disorders in STZ-induced diabetic rats were also attenuated after intake of *Akkermansia muciniphila* [131]. Furthermore, enriched *Blautia* may contribute to the amelioration of inflammation and insulin resistance in diabetes via the production of short-chain fatty acids [132].

In a previous study, D-fagomine was proved to reverse sucrose-induced steatosis and hypertension, presumably by reducing the postprandial levels of fructose in the liver [66]. Lately, Ramos-Romero et al. measured the effect of buckwheat iminosugar D-fagomine on diet-induced pre-diabetes in rats. They found that D-fagomine could reduce fat-induced impaired glucose tolerance, inflammation markers, and mediators. In addition, the changes in the main two bacterial phyla, *Bacteroidetes* and *Firmicutes*, in the intestinal tract were measured, and D-fagomine had no impact on modifying the reduction in the *Bacteroidetes*/*Firmicutes* ratio that led to body weight gain. Moreover, compared to the effect of D-fagomine on fat-induced changes in *Enterobacteriales* and *Bifidobacteriales*, it had a significant effect on glucose tolerance and inflammation [133].

In brief, obesity is recognized as the main cause of diabetes; hence, the pathophysiological parameters and changes in gut microbiota, such as the weight loss trend, insulin resistance, glucose intolerance, and inflammation markers would be expected to be similar. The composition of gut flora also exhibited similar alterations with an increasing abundance of *Blautia* and *Akkermansia* and a reduction in the numbers of *Lachnospiraceae* and *Faecalibaculum*. However, the relationships among gut microbiota, obesity, and diabetes are still unclear.

## 6. Prebiotic Effect

The concept of prebiotics was proposed by Gibson and Roberfroid in 1995. They first defined a prebiotic as a “non-digestible food ingredient that beneficially affects the host by selectively stimulating the growth and/or activity of one or a limited number of bacteria in the colon, and thus improves host health” [134]. The functional characteristics of prebiotics include resistance to low pH in the stomach, resistance to hydrolysis by mammalian enzymes, resistance to absorption in the upper gastrointestinal tract, ability to be fermented by intestinal microflora, and selective stimulation of the growth and/or activities of intestinal bacteria related to host health and overall well-being [13,134]. Generally, non-digestible carbohydrates were considered to be the main prebiotics, but other compounds, including phenolics, polyunsaturated fatty acid, linoleic acid, and phytochemicals, were included in the updated prebiotic definition [135]. As our knowledge and understanding of the gut microbiota and the importance of the gut microbiota increases, consumers must understand the key differences between the different forms of prebiotics and their presence in a variety of foods and related products.

Whole grains are rich in dietary fiber, micronutrients, and phytochemicals, which are considered to be natural prebiotics. For instance, the effect of oat bran on the human fecal microbiota composition was measured via in vitro anaerobic batch-culture experiments. The 16s rRNA sequencing results showed that the populations of *Proteobacteria* and *Bacteroidetes* were increased after oat supplementation, whereas fermentation of the 1% (*w*/*v*) oat bran increased the abundances of *Bifidobacterium* unassigned at 10 h and *Bifidobacterium adolescentis* at 10 and 24 h, compared to the control group. The SCFA analysis showed that consumption of oats stimulated the production of acetic and propionic acid, while oat bran fermentation resulted in an elevated SCFAs content. Meanwhile, β-glucan treatment induced an increase in the phylum *Bacteroidetes*, and the polyphenol mix induced an increase in the *Enterobacteriaceae* family at 24 h. It was concluded that the prebiotic effect of oats could be attributed to the synergy of all oat compounds within the complex food matrix [136]. Likewise, the prebiotic effect of quinoa and amaranth were evaluated in batch cultures with fecal human inocula. In this study, fluorescence in situ hybridization (FISH) was used to explore the microbiota composition and metabolic products. After treatment, the changes in the SCFAs of the quinoa group and the amaranth group were consistent with the decrease in pH. Moreover, there were significant alterations in the gut flora, including *Bifidobacterium spp.*, *Lactobacillus–Enterococcus*, *Atopobium*, *Bacteroides–Prevotella*, *Clostridium coccoides–Eubacterium rectale*, *Faecalibacterium prausnitzii*, and *Roseburia intestinalis*. Due to the growth of these bacterial groups, the SCFAs, acetate, propionate, and butyrate were markedly increased, which is critical for maintaining a balanced intestinal microbiota [137].

The Maillard reaction (MR) produces several low-mass molecules by reducing sugar and amino acids during food heat treatment and storage; these low-weight compounds are usually recombined by a series of advanced MRs to form melanoidins [138]. Because they have a similar structure to fibers, melanoidins were considered to have prebiotic properties [139]. Aljahdali and co-workers examined the effect of intake of melanoidins of barley malts on gut microbiota in mice. It was found that the melanoidins significantly changed the gut microbiota and stimulated the production of SCFAs. The populations of *Lactobacillus*, *Parasutterella*, *Akkermansia*, *Bifidobacterium*, and *Barnesiella* were remarkably increased, while *Dorea*, *Oscillibacter*, and *Alisitpes* were decreased, proving the prebiotic effect of melanoidins [140].

## 7. Effect on Other Chronic Diseases

### 7.1. Metabolic Syndrome

Metabolic syndrome is a complex disease with a rapid increase in incidence in recent years, and dietary intervention is an effective approach to ameliorate the metabolic status of patients [141]. Dietary composition can actively change the composition and function of intestinal microflora, so as to improve individual conditions related to metabolic disorders [142]. Barley β-glucans have been demonstrated to exert a hypoglycemic effect and a cholesterol-lowering effect. In an experiment by Velikonja et al., the impact of a dietary supplement of barley β-glucans on metabolic syndrome patients was investigated. After treatment, TC in plasma was decreased compared to the control group. Moreover, the fecal SCFAs were markedly changed, with increased propionic acid, while the acetic acid was decreased in the control group. Furthermore, 16s sequencing results showed that the populations of health-associated *Bifidobacterium spp.* and *Akkermansia municiphila* were increased after barley β-glucans consumption [143].

Growing evidence suggests that circadian disruption may increase the risk of metabolic diseases, causing a series of metabolic abnormalities including obesity, dyslipidemia, and glucose intolerance that are regarded as circadian-disruption-induced metabolic syndrome (CDIMS). Moreover, circadian disruption can increase the gut permeability, leading to dysfunction of the gut barrier and alteration of the gut microbiota [144]. In an induced CDIMS mice model, intake of oat β-glucans mitigated CDIMS, resulting in an increase in body weight, liver-weight-to-body-weight ratio, and plasma leptin concentration, and restored glucose tolerance. The analysis of gut microbiota showed that oat β-glucans increased the species richness and improved the abundance of seven bacterial genera and increased butyrate producers such as *Ruminococcaceae* and *Lachnospiraceae*, which contribute to glucose homeostasis and gut barrier protection [145]. On the other hand, antibiotic overuse can also destroy the gut microbiota by changing the community structure and reducing the diversity, resulting in gut barrier dysfunction [146,147]. Zhu and co-workers assessed the effect of a water-soluble polysaccharide from Fagopyrum esculentum Moench bee pollen (WFPP) on gut barrier integrity in antibiotic-treated mice. WFPP reversed the growth retardation, atrophy of the thymus and spleen, increased gut permeability, and intestinal barrier damage under ceftriaxone (a broad-spectrum antibiotic) treatment. Furthermore, WFPP was correlated with reduced inflammatory response and enhanced intestinal sIgA secretion. In addition, 16S rDNA sequencing results showed that WFPP ameliorated gut microbiota dysbiosis induced by ceftriaxone, and especially that it regulated the populations of *Proteobacteria* (sIgA-secretion-related bacteria) and *Enterococcus* (inflammation-related bacteria) [148].

### 7.2. Neurodegenerative Disorders

Neurodegenerative disorders such as Alzheimer’s disease (AD) and dementias are major causes of incidence rates, reduced quality of life and medical costs in elderly people [149]. There is evidence that microbial changes are related to neuroinflammation and cognitive impairment, which are two critical features of AD pathogenesis and progression [150]. Although neurodegenerative diseases are currently incurable, one third of dementia cases can be prevented by addressing lifestyle factors, including diet [149]. As an example, β-glucan prevents cognitive impairment caused by a high-fat and fiber-deficient diet (HFFD), as shown by behavioral appraisement through object positioning, new object recognition and nesting tests. The results showed that β-glucan reversed the cognitive function loss through more optimized synaptic and signaling transduction pathways in crucial brain areas, while intake of β-glucan also improved gut barrier dysfunction and reduced bacterial endotoxin translocation. Moreover, β-glucan alleviated gut microbiota dysbiosis caused by HFFD, especially *Bacteroidetes* at phylum level and its lower taxa [151]. A previous study demonstrated that the phylum *Bacteroidetes* was intimately related to neurodegenerative diseases. There was a lower abundance of *Bacteroides* at genus level in the gut microbiota of dementia patients, whereas the population of *Bacteroides fragilis* at species level was lower in patients with cognitive impairment [152,153]. According to the authors, oat β-glucan increased satiety in diet-induced obese mice by activating the gut–hypothalamic (PYY-NPY) axis [154]. SCFAs that were produced from β-glucan fermentation efficiently increased PYY production in human entero-endocrine cells [155]. In addition, excessive dietary energy intake and insulin resistance have adverse effects on human cognition, but energy restriction could enhance neural plasticity by reducing inflammation and increasing the expression of synaptic-plasticity-related proteins [156]. Therefore, β-glucan consumption is important for preventing cognitive impairment induced by HFFD by maintaining the gut microbiota–brain axis.

### 7.3. Malnutrition

Malnutrition is usually caused by insufficient protein and micronutrients due to lack of food [157]. Due to the poorly developed immune system and delayed puberty, children are the group most vulnerable to malnutrition. Recently, Li and co-workers evaluated the effect of a millet supplement on malnutrition and gut microbiota dysbiosis in a malnourished pig model. Compared to the control group, the millet supplement improved the growth status, gastrointestinal pathology, and content of total protein and globulin in the blood. Moreover, intake of millet increased the abundance of *Bacteroidetes*, *Firmicutes*, and *Lachnospira spp.*, while the population of *Proteobacteria* was decreased. Furthermore, a Pearson’s correlation analysis showed a strong positive correlation between the abundances of *Faecalibacterium* and *Lachnospira spp.* and body weight, crown–rump length and total serum protein [158]. Previously, a low abundance of *Faecalibacterium prausnitzii* was found in the intestines of malnourished children and malnourished pigs in Bangladesh [159]. Moreover, the abundance of *Lachnospira spp.* was positively associated with an anti-inflammatory effect in weaner pigs fed with an HFD [160]. Therefore, millet exhibited a promising benefit in regulating gut microbiota dysbiosis in malnourished pigs.

## 8. Prospects

In this study, we reviewed the functional components of coarse grains in alleviating chronic diseases through modulating the gut microbiota. A correct understanding of the critical role of coarse grains in gut health and microbial regulation could help ameliorate chronic diseases and improve quality of life. The complexity of mechanisms among the components of coarse grains, the digestive system and the gut microbiota is an important factor restricting development and utilization of coarse grains. Despite the fact that many bioactive substances in coarse grains have been proved to serve various functions in both in vitro and in vivo experiments, there is still only limited research on accurate metabolic processes of bioactive substances and their functional signal pathways. Moreover, the intensity and the targeting effects of these substances in the treatment of chronic diseases are far less than those of drugs. At present, there are a number of mature plant-derived drug evaluation methods, including in vitro high-throughput screening, animal model experiments, and human trials. However, single-substance experiments are not suitable for assessing the nutritional characteristics of coarse grains.

Generally, coarse grains are considered to be food rather than drugs. Therefore, coarse grains play a preventive and regulatory role via the synergistic action of various substances. Moreover, gut dysbiosis usually occurs in the early stage of disease. In order to ensure that the active substances of coarse cereals play a more effective role in improving chronic diseases, several aspects should be further studied in detail, including: (1) improving the bioactivity of these substances, which is effective and important for further research and application; (2) exploring more precise and persuasive experiments to clarify the interaction mechanisms between gut microbiota and bioactive substances; (3) combining the in vitro bioreactor research model and the in vivo gut reaction model to establish rapid screening and comprehensive evaluation of the bioactivity of coarse cereals. The outcome of this research would be helpful to further clarify the beneficial effects of cereal bioactive components on chronic diseases.
